# Being a role model for preventive action

**DOI:** 10.3389/fpubh.2026.1973597

**Published:** 2026-10-26

**Authors:** Conny Seeleman, Nienke van den Berg, Anna Kersten, Marielle Jambroes

**Affiliations:** Department of Global Public Health and Bioethics, Julius Center, Universitair Medisch Centrum Utrecht, Utrecht, Netherlands

**Keywords:** health professionals, medical education, preventive action, public health, role model, students

## Abstract

This article highlights the crucial role of clinicians as role models in fostering preventive action within medical education. Role modeling is a powerful mechanism in workplace learning, contributing both to medical students' professional identity formation and to their self-efficacy through observational (vicarious) learning. Rather than learning from a single exemplary physician, medical students construct a “mosaic” of role models, drawing on diverse professionals who each demonstrate different competencies. This underscores the importance of exposing medical students to varied clinical perspectives. Despite the growing importance of prevention—driven by aging populations, increasing multimorbidity, and health inequities—medical students report limited exposure to role models who explicitly demonstrate preventive care. Preventive actions are often underrepresented or remain implicit in daily clinical practice, partly because curative care is prioritized and population-level activities are less visible. Additionally, clinicians may not recognize or articulate their own preventive efforts as role modeling opportunities. To address this gap, the article introduces a framework describing preventive action at both individual and population levels, structured around three steps: identification, intervention, and evaluation. Building on this, it outlines how clinicians can model preventive care effectively using four phases of role model learning: attention, retention, reproduction, and motivation. Practical strategies include making preventive actions visible, explaining underlying reasoning, creating opportunities for practice, and demonstrating outcomes. The article concludes that deliberate and explicit role modeling of prevention, combined with active student engagement, can strengthen learning and support the integration of preventive care as a core component of medical professionalism.

## 1 Introduction

Every physician serves as a role model in clinical practice. Role modeling is a powerful educational mechanism in workplace learning because it contributes to both professional identity formation and self-efficacy (1–3). Through observing physicians in practice, medical students gradually learn what it means to think, act and feel like a physician, thereby shaping their professional identity. Role models also strengthen self-efficacy through vicarious learning: by observing how experienced physicians perform professional tasks and manage challenges, medical students gain confidence in their own ability to carry out these behaviors. In clinical settings, observing role models, discussing their actions, and reflecting on outcomes further supports the development of self-efficacy, which strongly influences whether students will perform these behaviors in practice themselves.

Recent research shows that medical students do not learn from one exemplary physician alone; instead, they assemble a role model from multiple individuals (4). Each professional offers different qualities that contribute to medical students' development. For educators, this can ease the pressure of feeling they must embody the “perfect” role model. At the same time, it underscores the importance of giving medical students access to a variety of professionals so they can gather enough “pieces” to build their mosaic-like role models.

Most role-model studies focus on qualities such as clinical expertise, professionalism and communication skills. Far less attention has been paid to how role models influence students' understanding of physicians' responsibilities in disease prevention and health promotion. This is increasingly important, as aging populations, multimorbidity and persistent health inequalities place prevention higher on the healthcare agenda (5–7).

Despite the recognized importance of prevention, medical students frequently report a lack of role models for preventive action (5). Preventive activities are often overshadowed by diagnostic or curative work or occur outside routine clinical encounters, making them largely invisible to medical students (8). Moreover, clinicians may not recognize their own preventive practices as opportunities for role modeling. Consequently, students have limited opportunities to observe prevention as an integral component of medical practice. This may hamper the development of their self-efficacy for preventive action, and because they do not see the outcomes of such actions in clinical practice, their motivation to engage in preventive action may further reduce. These findings suggest that clinical teachers should always be aware of their role as role models–not only in clinical decision-making and professional behavior, but in across all CanMEDS roles, including health advocacy (9).

Understanding how preventive role modeling occurs is increasingly important if medical education aims to prepare future physicians for growing societal challenges related to chronic disease, multimorbidity and health inequities. Therefore, this paper explores how clinicians can become effective role models for preventive action. Specifically, it discusses (1) preventive action at both the individual and population level and (2) the application of the four phases of learning from role models to preventive care.

## 2 What does preventive action involve?

Many scholars argue for stronger attention to prevention and population health in medical curricula. Within medical education, this is reflected in the health advocacy role—one of the seven CanMEDS competencies physicians are expected to demonstrate (8). In the Netherlands, learning objectives for prevention have been defined, but they do not specify which activities are included in preventive practice. Professional responsibilities in prevention also differ across healthcare systems (10).

In our recent project *Prevention in the Medical Curriculum*, we developed a framework that clarifies which actions constitute preventive practice (11). The framework supports student learning and enables clinicians to recognize preventive actions in their own work.

Table 1 presents the framework, outlining preventive tasks at both the individual patient level and the population level. These tasks can be applied broadly across healthcare settings. Each level includes three process steps: identification, intervention, and evaluation.

**Table 1:** Framework of preventive tasks at individual and population level.

	**At the individual level** With an individual person, often in the consultation room.	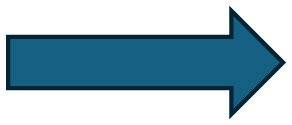 Knowing when action is required at the population level.	**At the population level** For a (sub)population, often outside the consultation room.
Identification and registration	Identify and register social determinants of health that affect an individual patient and recognize opportunities for prevention.		Identify a health risk within a (sub)population based on experiences in practice and verify this with colleagues and data.
Intervention	Engage in conversation, advise, and guide patients toward healthy behaviors, or refer them when necessary.		Address identified health issues in appropriate arena and consider one's own role as a physician and the role of potential collaborators in this matter.
Evaluation	Evaluate the impact of the actions taken.		Evaluate the effect of the actions taken and share findings with relevant partners or through scientific channels.

The framework illustrates that preventive action extends beyond the consultation room and includes attention to population health. Clinicians are uniquely positioned—in the consultation room and in hospital wards—to recognize not only individual health problems but also emerging health risks within specific groups. For example, emergency physicians may notice an increasing number of patients with head injuries from e-bike accidents, or neurologists may observe young people presenting with serious adverse neurological effects related to nitrous oxide use. In many cases, broader societal health issues first become visible and are documented in the clinical setting. See box 1 for an example (Box 1).

Box 1 Case study.

**Prevention at population level: an example from the Netherlands**

In 2017, following several severe dog-bite incidents, calls were made to introduce a ban on fighting dogs. However, the extent of the problem was unclear due to the lack of systematic registration of bite incidents. In response, the Dutch Society for Plastic Surgery (NVPC) initiated a 12-month registration of patients with severe dogbite injuries treated by plastic surgeons. Over the course of 1 year, they recorded 70 cases of serious injuries; 50% of the victims were children or infants. These findings were published in a Dutch professional journal and subsequently picked up by the media.

The physicians involved argued that it was now the government's responsibility to act. This led to a parliamentary proposal to ban fighting dogs, and a national dog-bite reporting center managed by the government.

There are also many examples of physicians taking action when health risks are identified. Consider physicians who advocated for vaccine equity during the COVID-19 pandemic, or the Dutch *Dokters in de Lead* network campaigning against smoking and the tobacco industry. Intervention at the population level is always carried out in collaboration with others. It involves addressing the identified health issue in the appropriate arena—such as public health agencies, professional organizations, or the public sphere. It is important to reflect on which role you can and wish to take, given your professional position, in contributing to the solution of these problems.

Finally, as in clinical care, it is essential to evaluate the impact of the actions taken and to share the outcomes with relevant partners or through scientific channels.

To become a role model for preventive action, the first step is to become aware of your own actions. Look beyond the obvious examples, such as lifestyle counseling in the consultation room, and consider prevention-related activities outside the clinical encounter, including team meetings, policy activities, professional association meetings or your contribution to discussions on population health issues.

Medical students should have the opportunity to observe and engage with physicians who demonstrate these preventive tasks, in addition to their theoretical education on prevention.

## 3 How to make role modeling for prevention explicit and effective?

It is helpful to understand how medical students learn from a role model. In the process of learning from a role model, four phases can be distinguished (12). Below, the four phases are described, and tips are provided on how they can be linked to preventive action.

### 3.1 Attention

Medical students need to *see* the behavior that educators and healthcare professionals want them to emulate. This means that medical students must pay attention to this behavior to recognize it and subsequently observe it in healthcare professionals in clinical practice. Therefore, healthcare professionals should make their preventive actions visible to medical students. For many healthcare professionals, prevention is not at the forefront of daily practice. Therefore, it is important that healthcare professionals reflect on how they incorporate preventive action in their own work. The framework presented in earlier paragraphs can be used to support this process. For example, healthcare professionals can explain how they identify red flags—such as financial stress or the lack of a social network—during a patient interview. They can tell them about important public health issues that are addressed within their professional association or about partnerships with organizations outside the hospital they are involved in.

### 3.2 Retention

Medical students need to be able to remember the observed behavior so that it *sticks*. At this stage, it helps medical students to understand the *why* behind certain behaviors. Therefore, healthcare professionals should engage medical students in discussions about the rationale underlying preventive actions. Discussing why healthy nutrition is promoted during hospital stays, or why professionals participate in initiatives to improve accessibility for patients with low health literacy, can help students understand the rationale behind preventive practice.

### 3.3 Reproduce

In this phase, medical students translate information about the new behavior into action. For this, it is important that opportunities are available to practice the new behavior. Medical students often need guidance to recognize such opportunities and to use them effectively for their learning process. Role models and teachers can support this by drawing medical students' attention to opportunities to practice preventive action. It is also important that they provide medical students with feedback on their behavior, to reinforce and improve the new behavior.

### 3.4 Motivation

Medical students need to be motivated to imitate and perform the behavior. Here, vicarious learning also plays an important role: medical students closely observe the effects of the behavior they witness, for example on clinical outcomes or through positive reactions from colleagues or patients. This influences their motivation to perform the behavior themselves. Therefore, it is important that medical students gain insight into the outcomes or consequences of the behaviour (1). In the case of preventive action, the effects are often not immediately visible in practice (for example, because they involve long-term outcomes). However, healthcare professionals can share their own experiences of preventive practice. In addition, professionals can demonstrate impact by, for example, referring to papers or newspaper articles that show the influence of public health working groups in which you participate. At the same time, preventive work is often constrained by invisible barriers, leaving limited time and funding. Medical students therefore need to learn to make well-considered decisions about which preventive tasks to undertake.

## 4 Discussion of practical implications

Medical education should deliberately incorporate and model preventive action in their daily work to be effective role models. However, medical students can be involved in this too, by asking them for feedback on what they have observed or to invite them to share good examples they have observed during other clinical rotations. In this way, learning about preventive action becomes a shared and collaborative process. Examples may include activities such as social prescribing, which make visible how healthcare professionals can address social determinants of health through preventive action. Although this paper focuses on medical education, the four-phase framework may also be relevant to other health professions in which preventive action and role modeling form part of workplace learning.

## Data Availability

The original contributions presented in the study are included in the article/supplementary material, further inquiries can be directed to the corresponding author.
